# Invited Letter to Editor in response to: Finland’s handling of selenium is a model in these times of coronavirus infections

**DOI:** 10.1017/S0007114520003839

**Published:** 2020-09-29

**Authors:** Giovanna Bermano, Catherine Méplan, Derry K. Mercer, John E. Hesketh

**Affiliations:** 1 Centre for Obesity Research and Education (CORE), School of Pharmacy and Life Sciences, Robert Gordon University, Aberdeen AB10 7GJ, UK; 2 School of Biomedical, Nutritional and Sport Sciences, Faculty of Medical Sciences, Newcastle University, Newcastle upon Tyne NE2 4HH, UK

The letter from Ulfberg and Stehlik suggests that the large difference in COVID-19 mortality rate between Sweden and Finland could be partly due to the Finns having a higher Se status due to the systematic supplementation of all agricultural fertilisers with Se since 1984^(1)^. This is an interesting suggestion and contrasts with the proposal that the Finns avoided high death rates by a combination of a strong lockdown, clear communication, quick action and the closing of borders^(2)^. It is likely that all these factors played a role in determining the outcome of COVID-19 infection, but we would support the view that Se status is also important. However, the preparedness of the Finnish health system and containment measures taken by Finland very much earlier during the spread of the infection differed greatly from the Swedish approach^(3)^, in which no strict lockdown was imposed on the population. Although Finland observed the lowest death rate of surrounding Scandinavian countries (other than Iceland), and Sweden the highest^(4)^, a comparison between Norway and Finland suggests a fairly similar mortality rate between the two countries (Table 1), despite the fact that the Norwegian population does not systematically receive Se-fortified food. Furthermore, data from USA and Canada, where the soil is widely naturally rich in Se and the Se status of the population is considered optimal or high^(5,6)^, show that in the USA, where no strict lockdown was implemented, the mortality rate was similar to the one identified in Sweden (0·0006), whereas in Canada the mortality rate was 0·0002, lower than Sweden and USA but higher than Finland (Table 1). It is clear that multiple factors determine the outcome from COVID-19 and so the potential relationship between Se status and mortality rates is complex. However, our view is that a combination of raising Se status and other public health measures may be effective in keeping mortality rates low.

**Table 1.** Mortality and percentage death rate in different countries

Country	COVID-19 cases* (cumulative total)	COVID-19 deaths* (cumulative total)	Population size†	Mortality	% Death rate (deaths/cases)
Canada	139 747	9193	37 806 632	0·00024	7
Denmark	20 571	633	5 796 391	0·00011	3
Finland	8750	339	5 542 378	0·00006	4
Germany	265 857	9371	83 783 942	0·00011	4
Iceland	2189	10	319 637	0·00003	0·5
Norway	12 393	265	5 429 519	0·00005	2
South Korea	22 783	377	51 278 058	0·00001	2
Sweden	87 575	5860	10 111 607	0·00058	7
USA	6 571 119	195 638	332 865 306	0·00059	3

* Values from the World Health Organization^(4)^.

† Values from https://www.worldometers.info/population/ (accessed September 2020).

Importantly, as highlighted by Ulfberg and Stehlik, after our article was accepted for publication, two cross-sectional studies from Germany and South Korea have been published^(7,8)^ in which Se status was measured in COVID-19 patients; both indicate a potential relationship between Se status and the severity of disease outcome. Despite the limitations associated with such explorative pilot studies, the relatively limited number of patients and samples, the lack of additional clinical data and the awareness that a decrease in Se biomarkers may be the result of an infection, both studies concluded that about 43 % of the COVID-19 patients had sub-optimal Se status. However, the Se status of the two cohorts was different, with a mean plasma Se level of 98·3 µg/l in the Korean patients and 50·8 µg/l in the German subjects. Interestingly, the difference in Se levels between these two small studies may reflect the differences observed at the population level (mortality rate sixteen times higher in Germany compared with South Korea and percentage death rates of 4 *v*. 2 %, respectively) (Table 1) and may further emphasise the importance of Se status in disease outcome. It is important to also stress that there has been a huge variability in the way data for both COVID-19 cases and death by COVID-19 have been reported in different countries and since the beginning of the pandemic.

The evidence linking Se and COVID-19 severity is, in our opinion, becoming significant when taken together with previous data linking other viral disease outcomes and Se intake^(9)^. Collaborative, integrated studies using material from COVID-19 patients, as well as mechanistic studies, are needed to confirm a link between Se status and COVID-19 disease severity so as to justify a supplementation trial to determine whether a large-scale Se supplementation, such as the one adopted by Finland, would reduce the mortality rate from SARS-CoV-2.

However, time is of the essence in combating this virus, especially as we near the winter influenza season. A key question is whether public health authorities should wait to get the proof of a link between Se status and COVID-19 outcome or go ahead and promote Se supplementation. The use of Se-enriched fertilisers has been successful in raising the Se status of Finns from a level regarded as sub-optimal to one above that found in many European countries including the UK. However, supplementation through fertiliser is regarded as relatively inefficient^(10)^ and would not become fully effective until the 2021 growing season, so dietary supplementation would be necessary to raise the Se status within next few months. We agree with Ulfberg and Stehlik in suggesting that public health authorities should consider promoting a modest Se supplementation as an additional, inexpensive and effective tool with which to fight COVID-19 and so lower risks of severe disease.

## References

[1] Alfthan G , Eurola M , Ekholm P , et al. (2015) Effects of nationwide addition of selenium to fertilizers on foods, and animal and human health in Finland: from deficiency to optimal selenium status of the population. J Trace Elem Med Biol 31, 142–147. 24908353 10.1016/j.jtemb.2014.04.009

[2] Puhakka J & McCarthy M (2020) Covid-19: what can we learn from Finland’s experience of the pandemic? BMJ Opinion. https://blogs.bmj.com/bmj/2020/09/04/covid-19-what-can-welearn-from-finlands-experience-of-the-pandemic/ (accessed October 2020).

[3] Habib H (2020) Has Sweden’s controversial covid-19 strategy been successful? BMJ 369, m2376. 32532807 10.1136/bmj.m2376

[4] World Health Organization (2020) WHO Coronavirus Disease (COVID-19) dashboard. https://covid19.who.int/table (accessed September 2020).

[5] Combs GF (2001) Selenium in global food systems. Br J Nutr 85, 517–547 11348568 10.1079/bjn2000280

[6] Johnson CC , Fordyce FM & Rayman MP (2010) Symposium on ‘Geographical and geological influences on nutrition’: factors controlling the distribution of selenium in the environment and their impact on health and nutrition. Proc Nutr Soc 69, 119–132. 19968907 10.1017/S0029665109991807

[7] Moghaddam A , Heller RA , Sun Q , et al. (2020) Selenium deficiency is associated with mortality risk from COVID-19. Nutrients 2, 2098. 10.3390/nu12072098 PMC7400921 32708526

[8] Im JH , Je YS , Baek J , et al. (2020) Nutritional status of patients with coronavirus disease 2019 (COVID-19). Int J Infect Dis 100, 390–393. 32795605 10.1016/j.ijid.2020.08.018 PMC7418699

[9] Bermano G , Méplan C , Mercer DK , et al. (2020) Selenium and viral infection: are there lessons for COVID-19? Br J Nutr, doi:10.1017/S0007114520003128. PMC7503044 32758306

[10] Haug A , Graham RD , Christophersen OA , et al. (2007) How to use the world’s scarce selenium resources efficiently to increase the selenium concentration in food. Microb Ecol Health Dis 19, 209–228. 18833333 10.1080/08910600701698986 PMC2556185

